# Noninvasive Mapping of the Electrophysiological Substrate in Cardiac Amyloidosis and Its Relationship to Structural Abnormalities

**DOI:** 10.1161/JAHA.119.012097

**Published:** 2019-09-09

**Authors:** Michele Orini, Adam J. Graham, Ana Martinez‐Naharro, Christopher M. Andrews, Antonio de Marvao, Ben Statton, Stuart A. Cook, Declan P. O'Regan, Philip N. Hawkins, Yoram Rudy, Marianna Fontana, Pier D. Lambiase

**Affiliations:** ^1^ Barts Heart Centre Barts Health NHS Trust London United Kingdom; ^2^ Institute of Cardiovascular Science University College London London United Kingdom; ^3^ The Royal Free Hospital UCL Hospitals Trust London United Kingdom; ^4^ Cardiac Bioelectricity and Arrhythmia Center Washington University in St Louis St. Louis MO; ^5^ MRC London Institute of Medical Sciences Imperial College London London United Kingdom

**Keywords:** amyloid, arrhythmia, electrophysiology mapping, imaging, T1 mapping, Electrophysiology, Magnetic Resonance Imaging (MRI), Electrocardiology (ECG)

## Abstract

**Background:**

The relationship between structural pathology and electrophysiological substrate in cardiac amyloidosis is unclear. Differences between light‐chain (AL) and transthyretin (ATTR) cardiac amyloidosis may have prognostic implications.

**Methods and Results:**

ECG imaging and cardiac magnetic resonance studies were conducted in 21 cardiac amyloidosis patients (11 AL and 10 ATTR). Healthy volunteers were included as controls. With respect to ATTR, AL patients had lower amyloid volume (51.0/37.7 versus 73.7/16.4 mL, *P*=0.04), lower myocardial cell volume (42.6/19.1 versus 58.5/17.2 mL, *P*=0.021), and higher T1 (1172/64 versus 1109/80 ms, *P*=0.022) and T2 (53.4/2.9 versus 50.0/3.1 ms, *P*=0.003). ECG imaging revealed differences between cardiac amyloidosis and control patients in virtually all conduction‐repolarization parameters. With respect to ATTR, AL patients had lower epicardial signal amplitude (1.07/0.46 versus 1.83/1.26 mV, *P*=0.026), greater epicardial signal fractionation (*P*=0.019), and slightly higher dispersion of repolarization (187.6/65 versus 158.3/40 ms, *P*=0.062). No significant difference between AL and ATTR patients was found using the standard 12‐lead ECG. T1 correlated with epicardial signal amplitude (cc=−0.78), and extracellular volume with epicardial signal fractionation (cc=0.48) and repolarization time (cc=0.43). Univariate models based on single features from both cardiac magnetic resonance and ECG imaging classified AL and ATTR patients with an accuracy of 70% to 80%.

**Conclusions:**

In this exploratory study cardiac amyloidosis was associated with ventricular conduction and repolarization abnormalities, which were more pronounced in AL than in ATTR. Combined ECG imaging–cardiac magnetic resonance analysis supports the hypothesis that additional mechanisms beyond infiltration may contribute to myocardial damage in AL amyloidosis. Further studies are needed to assess the clinical impact of this approach.

**Clinical Perspective**

**What Is New?**

- This is the first combined assessment of the electrical and structural ventricular substrate in cardiac amyloidosis patients, integrating data from advanced cardiac magnetic resonance imaging with recently proposed ECG imaging.
- This study demonstrates for the first time an interaction between cardiac magnetic resonance imaging indices (T1, T2, and extracellular volume) and ECG imaging features (signal amplitude, activation, and repolarization heterogeneity), and results suggest that several structural and functional parameters are different in AL and ATTR patients and could be used to discriminate between these 2 groups.

**What Are the Clinical Implications?**

- This novel approach may provide a better understanding of the underlying pathophysiology and enable more accurate prognosis in cardiac amyloidosis as well as track treatment effects, but further studies are needed to fully determine its clinical implications.

Cardiac amyloidosis is a disorder characterised by the deposition of abnormal protein fibers known as amyloid fibrils that can lead to heart failure and sudden cardiac death. Electrophysiological abnormalities and their relation to structural pathology are not well characterized in this population despite the fact that amyloid infiltration can induce complete heart block and has been linked to ventricular arrhythmias.^1^, ^2^, ^3^ The most common forms of cardiac amyloidosis are due to precursor proteins transthyretin (ATTR)^4^ and immunoglobulin‐derived light chains (AL),^5^ with cardiac involvement being the main driver of prognosis in both.^6^ Amyloid infiltration in cardiac ATTR amyloidosis is usually more severe than in AL, resulting in higher left ventricular (LV) mass and more severe systolic and diastolic dysfunction in ATTR.^7^ However, in AL amyloidosis the survival is significantly worse, with median survival from presentation of about 6 months as opposed to 6 years in ATTR,^6^ and the rate of sudden cardiac death is significantly higher, occurring in a third of cases within the first 90 days.^8^ This divergence between phenotypic severity and outcome remains poorly understood but has been ascribed to additional toxic effects of AL amyloid^9^ or to a faster rate of amyloid deposition in AL compared with ATTR,^8^ leading to more severe myocardial damage in AL.

Cardiac magnetic resonance (CMR) has been proven to be able to measure the continuum of amyloid infiltration and the myocardial response, both in terms of myocyte loss/myocyte hypertrophy and myocardial edema, providing a deeper understanding of disease pathogenesis and of the differences between the 2 main types of amyloidosis.^7^, ^10^, ^11^, ^12^, ^13^, ^14^ However, neither the ventricular electrophysiological (EP) substrate associated with cardiac amyloid deposition nor the potential EP differences between AL and ATTR have ever been characterized, leaving a critical knowledge gap in disease understanding. Electrocardiographic imaging (ECGI)^15^, ^16^ is a noninvasive technology that computes epicardial extracellular potentials and provides insight into potential arrhythmogenic substrates.^17^, ^18^, ^19^, ^20^ We conducted an exploratory CMR‐ECGI study to investigate for the first time the interaction between EP and structural abnormalities associated with cardiac amyloidosis and to determine how these may differ between AL and ATTR.

## Methods

The data that support the findings of this study are available from the corresponding author on reasonable request.

### Study Population

Twenty‐one patients with cardiac amyloidosis (11 AL and 10 ATTR) were prospectively recruited and underwent ECGI and CMR studies at The Royal Free Hospital, University College London Hospitals, United Kingdom. Cardiac ATTR amyloidosis was defined as the combination of symptoms with an echocardiogram consistent with or suggestive of cardiac amyloidosis,^21^ a grade 2 or 3 cardiac uptake on ^99m^Tc‐DPD (3,3‐diphosphono‐1,2‐propanodicarboxylic acid) scintigraphy in the absence of a monoclonal gammopathy, or, in the presence of monoclonal gammopathy, a cardiac biopsy confirming ATTR.^22^ All subjects underwent sequencing of exons 2, 3, and 4 of the *TTR* gene. Of the 10 ATTR patients, 8 were wild type, and 2 had hereditary ATTR associated with the *V122I* variant. Cardiac AL amyloidosis was determined on the basis of international consensus criteria^23^ as well as the combination of typical features on CMR and biopsy‐proven systemic AL amyloidosis on cardiac or noncardiac biopsy.^22^ All enrolled patients provided informed consent to participate in the research. The study was approved by the National Research Service Committee, London (14/LO/0360). Patients’ age, sex, etiology, and medication(s) are reported in Table S1.

Two groups of healthy volunteers were recruited: 1 served as control for CMR parameters (n=25 patients), and the other served as control for 12‐lead ECG and ECGI parameters (n=30 patients).

### ECGI Protocol and Analysis

**Figure 1:**
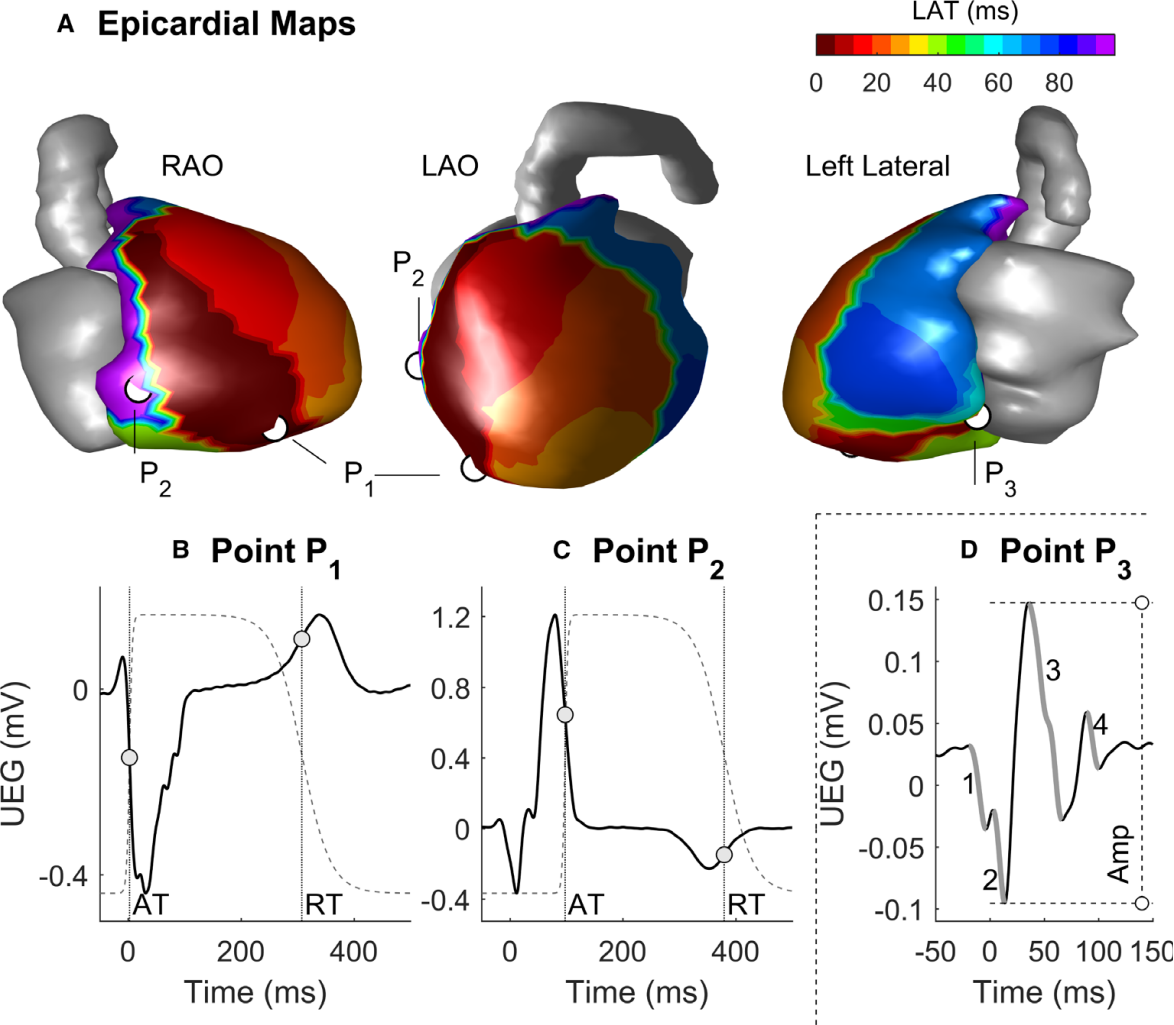
Representative example of epicardial potentials in cardiac amyloidosis reconstructed by ECGI. **A**, Local activation time (LAT) map in different angiographic views. **B** and **C**, Unipolar electrograms (continuous lines) from cardiac sites P_1_ and P_2_. Dots represent activation and repolarization times, and dashed lines represent local action potentials (schematic, for illustration purposes only). **D**, Unipolar electrogram from cardiac site P_3_ exhibiting fractionated QRS complex with 4 negative deflections (bold gray line) and low voltage. Amplitude (Amp) is measured as the difference between the maximum and minimum values of the unipolar electrogram within the QRS complex (vertical dashed line). AT indicates activation time; ECGI, ECG imaging; LAO, Left Anterior Oblique; RAO, Right Anterior Oblique; RT, repolarization time; UEG, Unipolar Electrogram.

ECGI provides noninvasive assessment of epicardial electrophysiology and has been described previously.^15^, ^20^ Briefly, 256 electrodes were placed to uniformly cover the patient's torso (ActiveTwo, BioSemi, Santiago, Chile), and body surface potentials were recorded at a sampling frequency of 2048 Hz for 5 minutes at rest in a supine position. Electrodes were then removed and replaced with magnetic resonance imaging opaque markers positioned in identical locations before a CMR scan was performed to acquire heart‐torso geometries. Data were exported and analyzed offline. Epicardial meshes and electrode locations on the torso were reconstructed using commercial software (Amira, ThermoFisher, Waltham, MA). Signal averaging was performed to enhance the signal quality of body surface potentials using custom software (Matlab, MathWorks, Natick, MA). Unipolar epicardial electrograms were reconstructed by solving the inverse problem of electrocardiography.^15^, ^16^, ^20^ Typically, ≈1000 electrograms were computed over the entire ventricular epicardium, with those over the valve plane excluded from analysis. At each cardiac site, activation (AT) and repolarization times (RT) were measured as the time of the steepest signal downslope (dV/dt_min_) during the QRS complex and the time of steepest signal upslope (dV/dt_max_) during the T‐wave, respectively. Cardiac intervals were referenced to the earliest AT and were carefully reviewed and corrected with semiautomatic custom software as in previous studies.^24^, ^25^, ^26^ Figure 1A shows a representative AT map from an ATTR patient, and panels B and C report examples of unipolar electrograms from 2 different sites. Activation‐recovery interval (ARI), a standard surrogate for action potential duration,^27^ was measured as ARI=RT–AT. Repolarization times were corrected for heart rate using the Fridericia formula. Global dispersion of AT, RT, and ARI (ΔAT, ΔRT, and ΔARI, respectively) was measured as the difference between the maximum and minimum value of AT, RT, and ARI, respectively, across the entire epicardium. Spatial gradients of activation (G_AT_) were measured as the absolute AT difference between neighboring sites divided by their distance, averaged across all neighbors.^20^ Spatial gradients of repolarization (G_RT_) were measured similarly. Epicardial signal amplitude was measured as peak‐to‐peak amplitude, ie, the difference between the maximum and the minimum (which can be negative) values within the QRS complex of the unipolar electrogram (Figure 1D). Signal fractionation, an indication of underlying abnormal electrical activity,^28^ was quantified by the number of negative deflections during the QRS complex (Figure 1D).

### CMR Protocol and Analysis

All participants underwent standard CMR on a 1.5 T scanner (Aera, Siemens Healthcare, Erlangen, Germany). A standard clinical scan protocol with late gadolinium enhancement imaging and T1 mapping (by Modified Look‐Locker Inversion recovery) before and after a bolus of gadolinium contrast (0.1 mmol/kg of gadoterate meglumine) was performed.^29^ Late gadolinium enhancement imaging was acquired using magnitude reconstruction and phase‐sensitive inversion recovery reconstruction in all patients. Postcontrast T1 mapping was performed 15 minutes after the administration of gadolinium. All CMR images were analyzed offline. Left ventricular volumes, mass, and function were analyzed using in‐house plugins on OsiriX, version 9.0.1 (Pixmeo SARL, Bernex, Switzerland). For T1 mapping, 4‐chamber and 3‐short‐axis maps (base, middle, and apex) were acquired. The 3‐short‐axis T1 maps were manually contoured at the endocardial and epicardial border, segmented into an American Heart Association 16‐segment model using the right ventricular insertion points. Hematocrit (h) was measured in all subjects immediately before each CMR study. As in previous studies,^7^, ^11^, ^14^ extracellular volume (ECV) was calculated as (1−h)×ΔR1_myocardium_/ΔR1_bloodpool_, where ΔR1 is the difference in relaxation rates (1/T1) precontrast and postcontrast, and total myocardial amyloid volume was calculated as LV mass/g×ECV, where g is the specific gravity of myocardium (1.05 g/mL). Total myocardial cell volume was calculated as LV mass/g×(1−ECV). For T2 mapping, 3‐short‐axis matching the T1 maps was acquired and analyzed in the same way as the T1 maps.

### Standard 12‐Lead ECG

A 12‐lead ECG was recorded, and standard parameters were derived. QRS amplitude was measured as the average of peak‐to‐peak QRS amplitude in limb, precordial, and all leads, respectively.^30^ Patients were classified as showing low QRS amplitude if the amplitude was <0.5 mV in all limb leads or <1 mV in all precordial leads. Additionally, Sokolow criteria,^31^ in which QRS low amplitude is defined as the sum of S‐wave in V1 and R‐wave in V5 or V6 lower than 1.5 mV, was also implemented.

### Statistical Analyses

Analyses of CMR and ECGI data were blind to cardiac amyloidosis pathology (AL and ATTR) but not to controls, as these patients were recruited at a further stage of the study.

Continuous variables are presented as median/interquartile range. The Wilcoxon rank‐sum test was used to test unpaired comparisons with statistical significance set at *P*<0.05. Given the exploratory nature of the study and the expected correlation among several variables, corrections for multiple comparisons were not performed.^32^ The statistical significance of the reported associations should be confirmed in future confirmatory studies. Correlation was assessed using the Spearman correlation coefficient. For variables presenting multiple values per patient (eg, 1 value per cardiac site or per anatomical segment), differences were evaluated between the mean values representing the within‐patients distribution. Correlations between EP and structural substrates were assessed by measuring the correlation coefficient between the mean ECGI and CMR parameters obtained by averaging over common anatomical segments (because ECGI and CMR provide epicardial and LV information, respectively, right ventricular segments were excluded from ECGI, and septal segments were excluded from CMR parameters). The standard 17‐segment model was used to identify common segments in the 2 modalities. Logistic regression was used to classify AL and ATTR patients. Univariate models were constructed using the subset of features that showed differences between AL and ATTR patients associated with *P*<0.10 (Wilcoxon rank sum test), and the least absolute shrinkage and selection operator method was used to identify the most suitable subsets of features to be tested in multivariate models. Cross validation was performed to assess possible issues related to overfitting using the leave‐1‐out technique. Accuracies achieved by different models were compared with the McNemar test.

## Results

Cardiac amyloidosis patients were predominantly male (81.8% of AL and 100% of ATTR), and ATTR were older than AL patients (80.5/7 versus 64/8.5 years, *P*=0.01, median/interquartile range).

### CMR Imaging Findings

**Figure 2:**
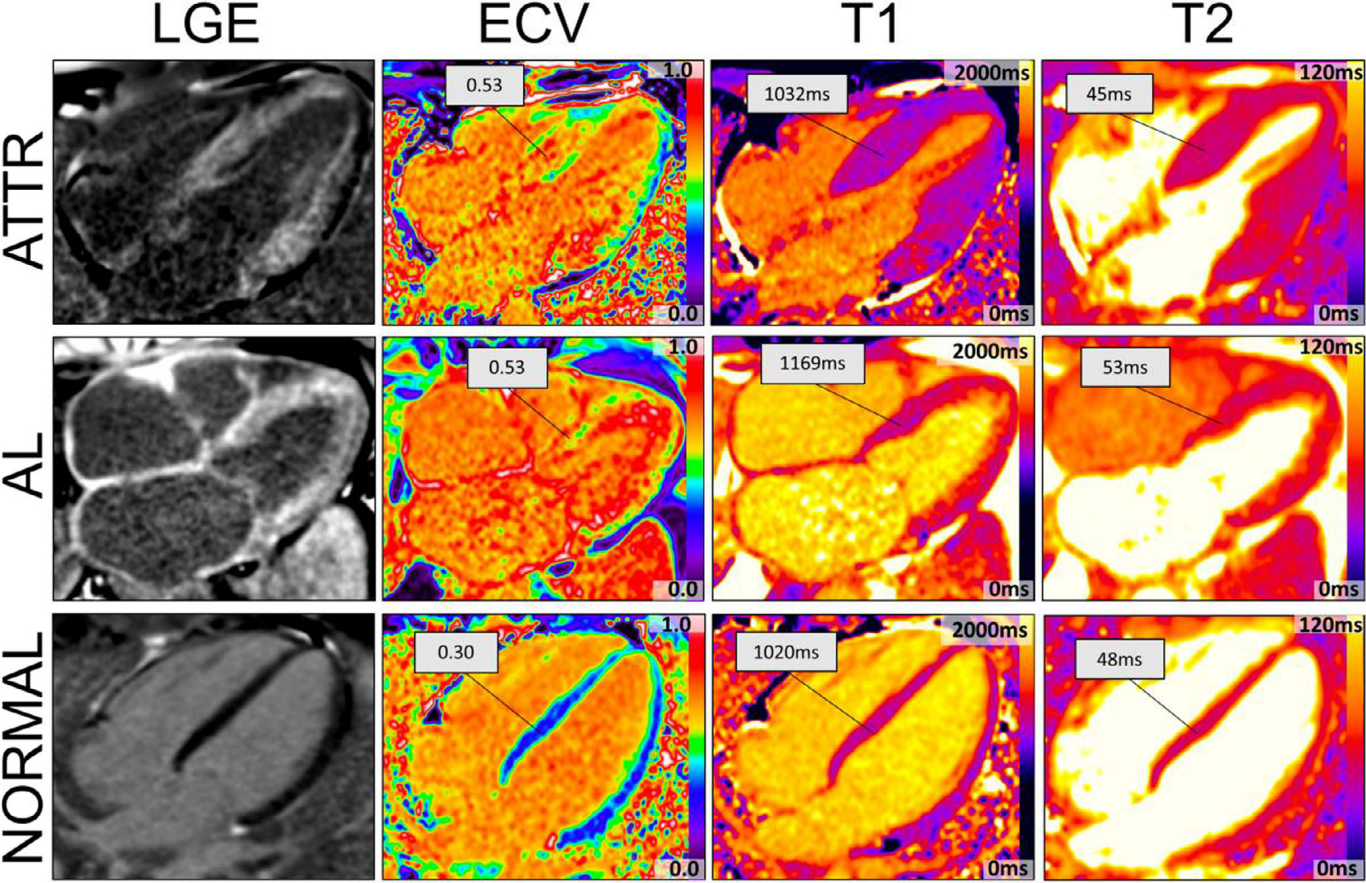
Late gadolinium enhancement (LGE), extracellular volume (ECV), native T1 and T2 maps for ATTR (top), AL (middle), and control (bottom) patients. ECV, T1, and T2 are higher in the 2 amyloid patients than in the control patient. T1 and T2 are higher in the AL than in the ATTR patient despite similar degree of cardiac amyloid infiltration (similar ECV). AL indicates light‐chain amyloidosis; ATTR, transthyretin amyloidosis; T1 and T2, magnetic fields in magnetic resonance imaging.

With respect to healthy controls, cardiac amyloidosis patients (both AL and ATTR) showed expected abnormalities, including higher ECV, T1, T2, LV mass, and diastolic impairment (Table 1). Differences were also observed between AL and ATTR patients. ECV was elevated in patients with both AL and ATTR amyloidosis (*P*=0.45, Table 1). LV mass (196/114.0 g versus 270/69.0 g, *P*=0.02), total myocardial amyloid volume (51.0/37.7 versus 73.7/16.4 mL, *P*=0.04), and myocardial cell volume (42.6/19.1 versus 58.5/17.2 mL, *P*=0.021) were higher in ATTR than in AL patients, whereas T1 (1172/64 versus 1109/80 ms, *P*=0.022) and T2 (53.4/2.9 versus 50.0/3.1 ms, *P*=0.003) were higher in AL than ATTR. LV ejection fraction was slightly lower in AL than in ATTR (58/13.8% versus 70/14.0%, *P*=0.06), mainly because of slightly larger end‐systolic volume in AL (57/31.8 mL versus 38/28.0 mL, *P*=0.06). Figure 2 shows late gadolinium enhancement, ECV, and T1 and T2 maps in patients with AL and ATTR amyloidosis as well as in a control patient.

**Table 1:** CMR Parameters Expressed as Median (Interquartile Range) or Number (Proportion)

CMR Parameters	Amyloid (n=21)	Controls (n=25)	*P* Value	AL (n=11)	ATTR (n=10)	*P* Value
Age, y	73.0 (19.2)	45.3 (12.6)	4.00E‐08^a^	64.0 (8.5)	80.5 (7.0)	1.1E‐02^a^
Male (n)	19 (90)	19 (80)	4.20E‐01	81.8	100	4.8E‐01
ECV (%)	51.6 (9.0)	0.3 (0.0)	2.30E‐07^a^	49.7 (7.8)	52.4 (11.3)	4.5E‐01
T1, ms	1155 (79)	989.8 (32.5)	1.60E‐08^a^	1172 (64)	1109 (80)	2.2E‐02^a^
T2, ms	52.6 (4.2)	48.0 (2.3)	8.40E‐06^a^	53.4 (2.9)	50.0 (3.1)	3.0E‐03^a^
LVEF, %	62 (16.5)	65.5 (6.0)	2.10E‐01	58 (13.8)	70 (14.0)	5.7E‐02
LVEDV, mL	138 (56.8)	175.5 (70.0)	8.10E‐03^a^	147 (66.5)	135 (28.0)	4.2E‐01
LVEDVi, mL/m^2^	71 (26.0)	87.5 (20.0)	7.00E‐03^a^	72 (34.5)	66 (20.0)	2.9E‐01
LVESV, mL	49 (33.8)	62.5 (33.0)	2.80E‐01	57 (31.8)	38 (28.0)	5.7E‐02
LVESVi, mL/m^2^	26 (18.8)	29.0 (10.0)	4.40E‐01	30 (12.5)	18 (14.0)	5.2E‐02
LV‐SV, mL	81 (29.0)	117.0 (32.0)	6.40E‐05^a^	80 (31.0)	82 (26.0)	8.3E‐01
LV‐SV‐i, mL/m^2^	41 (10.3)	57.5 (10.0)	2.60E‐05^a^	41 (9.8)	42 (10.0)	8.9E‐01
LV mass, g	250 (97.1)	117.0 (55.0)	2.00E‐06^a^	196 (114.0)	270 (69.0)	2.2E‐02^a^
LV mass‐i, mL/m^2^	127 (40.8)	58.5 (20.0)	2.60E‐07^a^	104 (51.3)	136 (24.0)	4.1E‐02^a^
TAPSE, mm	15.0 (10.3)	···	···	13.0 (15.0)	15.5 (6.0)	9.2E‐01
Total amyloid volume, mL	68.1 (29.5)	31.9 (11.9)	1.50E‐04^a^	51.0 (37.7)	73.7 (16.4)	4.5E‐02^a^
Total cell volume, mL	50.5 (20.4)	83.2 (40.1)	9.00E‐05^a^	42.6 (19.1)	58.5 (17.2)	2.1E‐02^a^

AL indicates light‐chain amyloidosis; ATTR, transthyretin amyloidosis; CMR, cardiac magnetic resonance; ECV, mean extracellular volume; EF, ejection fraction; ESV and EDV, end‐systolic and ‐diastolic volume, respectively; LV, left ventricle; SV, stroke volume; TAPSE, mean tricuspid annular plane systolic excursion; indices followed by “‐i” are indexed to body surface volume.

a *P*<0.05.

### Surface ECG Findings

Standard surface ECG analysis showed that with respect to controls, cardiac amyloidosis patients had wider QRS (106/17 versus 88/12 ms, *P*<0.005), longer PR interval (185/55 versus 160/26 ms, *P*<0.005), longer QTc (444/38 versus 415/28 ms, *P*=0.008), and lower QRS amplitude in all lead configurations (Table 2). The interval from the end of the QRS to the end of the T wave (measured as QTc–QRS) was not significantly different in cardiac amyloidosis patients as compared with control patients (*P*=0.73), suggesting that QTc prolongation could be explained by a widening of the QRS complex. T‐wave inversion and poor R‐wave progression were more frequent in cardiac amyloidosis than in controls. No surface ECG parameter showed statistically significant differences between AL and ATTR patients (Table 2). Because differences in EP parameters may be explained by age (73/19 years in cardiac amyloidosis versus 31/31 years in controls, *P*<0.005), a secondary analysis was performed by comparing 10 cardiac amyloidosis patients (5 ATTR+5 AL) with 10 age‐matched controls. Results were in agreement with those between amyloidosis patients and younger volunteers with the exception that although median QTc was still longer in cardiac amyloidosis, this difference was no longer significant (Table S2).

**Table 2:** Twelve‐Lead ECG Parameters

Surface ECG Parameters	Amyloid (n=21)	Controls (n=30)	*P* Value	Amyloid AL (n=11)	Amyloid ATTR (n=10)	*P* Value
AF (n)	3 (14.3%)	0 (0.0%)	6.39E‐02	1 (9.1%)	2 (20.0%)	1.00E+00
RR, ms	886 (187.5)	938 (126.5)	2.75E‐01	822 (219.0)	897 (164.0)	6.20E‐02
QRS, ms	106 (17.0)	88 (12.0)	3.03E‐05^a^	104 (14.0)	107 (20.0)	6.70E‐01
PR, ms	185 (55.0)	160 (26.5)	9.35E‐04^a^	185 (22.0)	199 (94.0)	6.00E‐01
QT, ms	444 (38.1)	415 (28.1)	7.59E‐03^a^	433 (65.0)	450 (27.6)	2.80E‐01
Poor R progression (n)	17 (81.0%)	1 (3.3%)	6.48E‐09^a^	11 (100.0%)	6 (60.0%)	3.51E‐02^a^
BBB (n)	3 (14.3%)	0 (0.0%)	6.39E‐02	2 (18.2%)	1 (10.0%)	1.00E+00
TW inversion (n)	8 (38.1%)	0 (0.0%)	3.20E‐04^a^	5 (45.5%)	3 (30.0%)	7.00E‐01
Amp‐limb, mV	0.40 (0.21)	0.95 (0.37)	1.99E‐07^a^	0.37 (0.21)	0.41 (0.37)	4.38E‐01
Amp‐precordial, mV	1.17 (0.46)	1.43 (0.37)	1.00E‐02^a^	1.02 (0.58)	1.22 (0.47)	2.05E‐01
Amp‐Sokolow, mV	1.20 (1.05)	2.45 (0.80)	9.74E‐07^a^	1.00 (0.75)	1.40 (1.10)	1.70E‐01
Low‐voltage limb (n)	11 (52.4%)	0 (0.0%)	7.41E‐06^a^	6 (54.5%)	4 (40.0%)	1.00E+00
Low‐voltage precordial (n)	2 (9.5%)	0 (0.0%)	1.65E‐01	2 (18.2%)	0 (0.0%)	4.76E‐01
Low‐voltage Sokolow (n)	13 (61.9%)	0 (0.0%)	4.27E‐07^a^	8 (72.7%)	5 (50.0%)	3.87E‐01

Differences between cardiac amyloidosis patients (AL+ATTR) and controls as well as between AL and ATTR patients. AF indicates atrial fibrillation; AL indicates light‐chain amyloidosis; Amp, amplitude; ATTR, transthyretin amyloidosis; BBB, bundle branch block; QT, QT interval corrected for heart rate (Fridericia formula); RR and PR, RR, and PR intervals, respectively; TW, T‐wave.

a Statistically significant *P*‐values.

### Ventricular EP Abnormalities in Cardiac Amyloidosis

**Figure 3:**
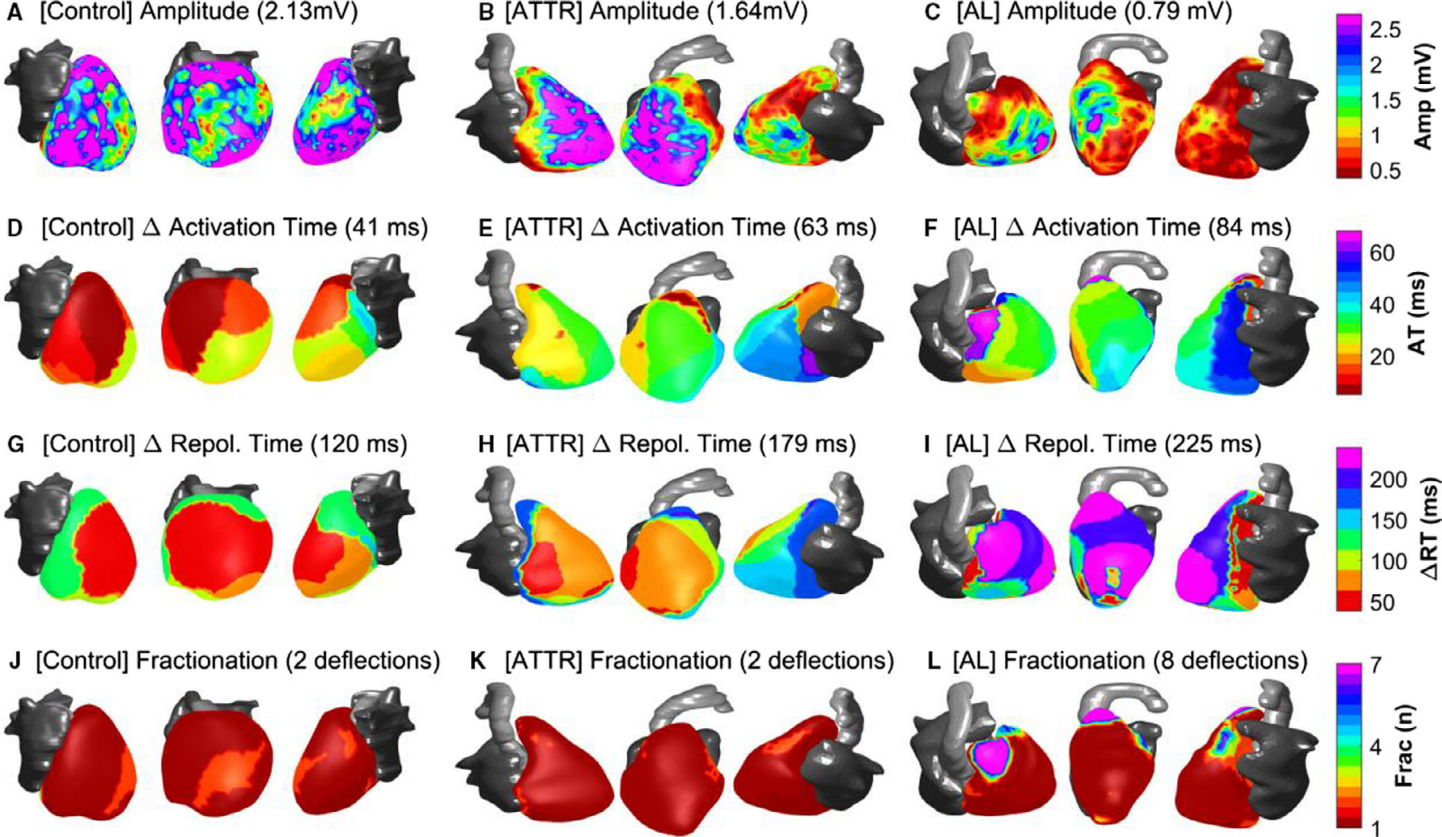
Example of a control (left), ATTR (center) and AL (right) patients showing marked differences in ECGI parameters, which are more pronounced in the AL group. **A** through **C**, Epicardial signal amplitude is lower in cardiac amyloidosis vs control and lower in AL vs ATTR. **D** through **F**, Total activation time (ΔAT) is longer in cardiac amyloidosis than in controls. **G** through **I**, Dispersion of repolarization (color coded as the difference between local and minimum repolarization time) is higher in cardiac amyloidosis vs control and higher in AL vs ATTR. **J** through **L**, Signal fractionation, measured as number of negative deflections in the QRS complex of the unipolar electrogram, is more elevated in AL patients. AL indicates light‐chain amyloidosis; Amp, amplitude; ATTR, transthyretin amyloidosis; ECGI, ECG imaging; Frac, fractionation; RT, repolarization time.

Figure 3 shows EP differences in representative control, AL, and ATTR patients. Cardiac amyloidosis patients presented multiple severe EP abnormalities (Table 3, Figure 3). With respect to healthy volunteers, epicardial signal amplitude was markedly reduced (1.15/0.82 versus 1.96/0.89 mV, *P*<0.001), intraventricular conduction was slower (total activation time equal to 65.4/24.7 versus 41.5/11.7 ms, *P*<0.001), and markers of conduction abnormality were higher (epicardial signal fractionation equal to 1.18/0.11 versus 1.10/0.10, *P*<0.001, spatial gradient of activation equal to 0.35/0.14 versus 0.23/0.08 ms/mm, all *P*<0.001). Furthermore, all repolarization parameters demonstrated a longer and more spatially dispersed repolarization process in cardiac amyloidosis than in controls (eg, ARI equal to 283.2/23.4 versus 242.6/24.8 ms, *P*<0.001, ΔARI=180.2/63.0 versus 1432/19.6 ms, *P*<0.001). In secondary analysis, comparison between cardiac amyloidosis patients (5 ATTR+5 AL) and 10 age‐matched controls showed similar results, with all differences remaining significant despite a reduction in statistical power (Table S3).

**Table 3:** ECG Imaging Parameters

ECGI Parameters	Amyloidosis (n=21)	Controls (N=30)	*P* Value	Amyloidosis AL (n=11)	Amyloidosis ATTR (n=11)	*P* Value
Age, y	73.00 (19.25)	31.45 (31.55)	9.11E‐07^a^	64.0 (8.5)	80.5 (7.0)	1.11E‐02^a^
HR, bpm	74.61 (20.71)	58.99 (10.86)	6.39E‐03^a^	78.4 (22.0)	67.4 (19.2)	3.42E‐01
Amp, mV	1.15 (0.82)	1.96 (0.89)	4.14E‐04^a^	1.07 (0.46)	1.83 (1.26)	2.65E‐02^a^
Frac (n)	1.18 (0.11)	1.10 (0.10)	1.97E‐04^a^	1.24 (0.12)	1.18 (0.18)	1.93E‐01
mFrac (n)	3.00 (1.00)	3.00 (1.00)	8.29E‐02	4.00 (1.75)	3.00 (1.00)	1.95E‐02^a^
ΔAT, ms	65.43 (24.66)	41.50 (11.72)	7.50E‐07^a^	65.9 (26.9)	60.3 (17.6)	8.33E‐01
AT, ms	30.78 (6.99)	22.25 (10.00)	4.39E‐05^a^	30.2 (10.3)	30.9 (7.3)	3.79E‐01
ΔRT, ms	166.92 (41.35)	129.22 (24.34)	4.04E‐05^a^	187.6 (65.1)	158.4 (39.9)	6.20E‐02
RT, ms	318.17 (33.91)	272.27 (18.84)	7.91E‐09^a^	310.8 (46.4)	320.6 (27.7)	4.18E‐01
ΔARI, ms	180.22 (63.00)	143.23 (19.64)	4.14E‐04^a^	192.0 (71.7)	162.1 (54.3)	1.49E‐01
ARI, ms	283.22 (23.41)	242.62 (24.79)	4.14E‐07^a^	281.9 (42.9)	288.1 (19.6)	6.99E‐01
G_AT_, ms/mm	0.35 (0.14)	0.23 (0.08)	1.48E‐06^a^	0.35 (0.13)	0.35 (0.14)	8.60E‐01
G_RT_, ms/mm	0.85 (0.32)	0.94 (0.22)	4.38E‐01	0.90 (0.33)	0.76 (0.33)	2.18E‐01

Repolarization parameters were corrected for heart rate. AL indicates light‐chain amyloidosis; Amp, mean epicardial signal amplitude; AT, RT, ARI, mean activation, repolarization, and activation‐recovery interval, respectively; ATTR, transthyretin amyloidosis; ECGI, ECG imaging; Frac, mean number of negative deflections in fractionated QRS complexes; G_AT_ and G_RT_, spatial gradients of activation and repolarization; HR, heart rate; mFrac, maximum number of deflections in fractionated QRS complexes; ΔAT, ΔRT, ΔARI, dispersion of activation, repolarization, and ARI, respectively.

a *P*<0.05.

### Ventricular EP Differences Between AL and ATTR

**Figure 4:**
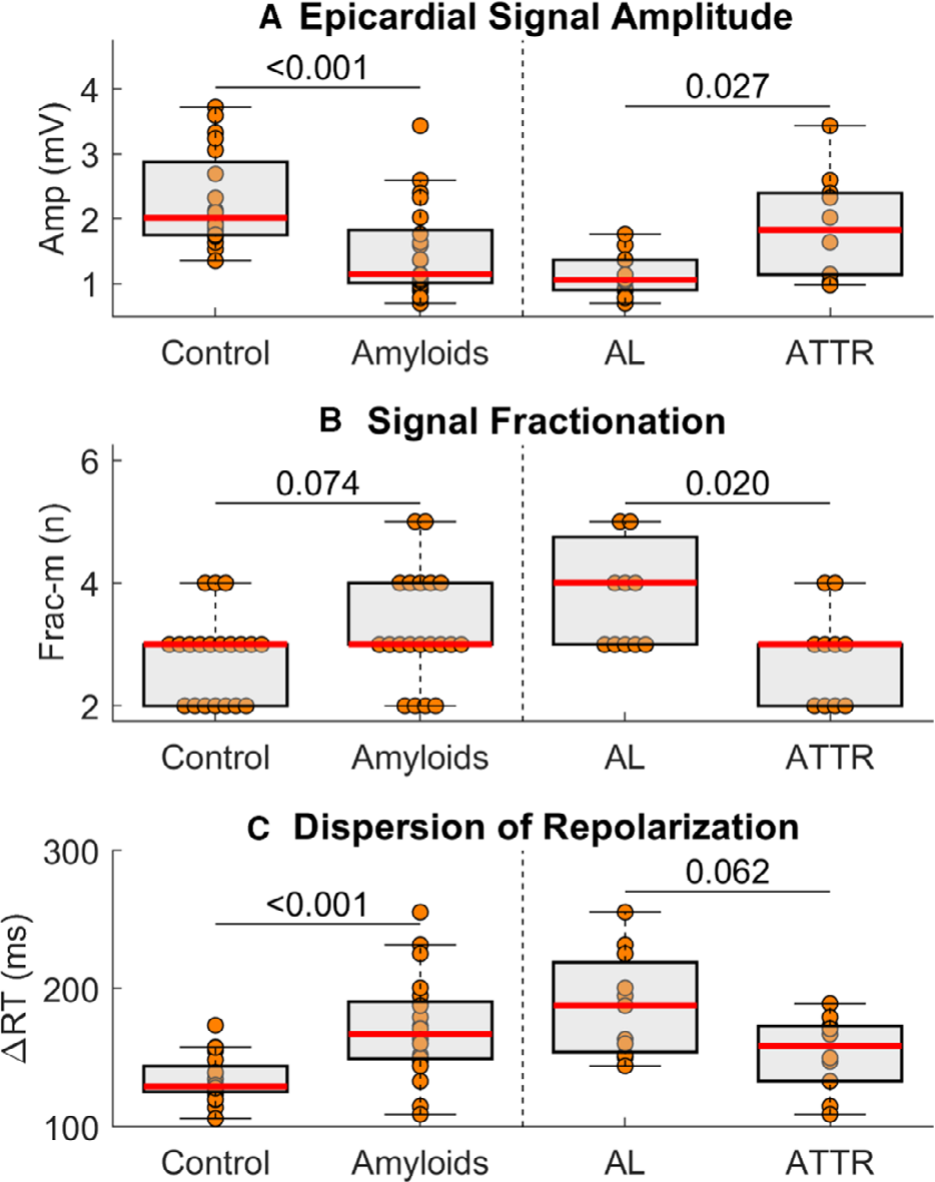
Pairwise comparison of 3 ECGI parameters (**A** through **C**) between controls and cardiac amyloidosis patients (ie, AL+ATTR patients, left) and cardiac AL and ATTR patients (right). Central line is the median, the edges of the box are the first (Q1) and third (Q3) quartiles, and the whiskers extend to the most extreme data points not considered outliers. *P*‐values are reported on top of the horizontal lines. AL indicates light‐chain amyloidosis; Amp, epicardial signal amplitude; ATTR, transthyretin amyloidosis; Frac, maximum number of negative deflections in fractionated epicardial signals; ΔRT, dispersion of repolarization.

As shown in the example reported in Figure 3, epicardial abnormalities were more pronounced in AL than in ATTR patients. Statistical analysis (Table 3) showed that AL patients had lower epicardial signal amplitude (Figure 4, 1.07/0.46 versus 1.83/1.26 mV, *P*=0.02), a higher degree of signal fractionation (Figure 4, maximum number of negative deflections equal to 4.00/1.75 versus 3.00/1.00, *P*=0.02), and longer (but nonsignificant) dispersion of repolarization (Figure 4, ΔRT equals 187.6/65.1 versus 158.4/39.9 ms, *P*=0.06) than ATTR patients.

### Interactions Between EP and CMR Parameters

**Figure 5:**
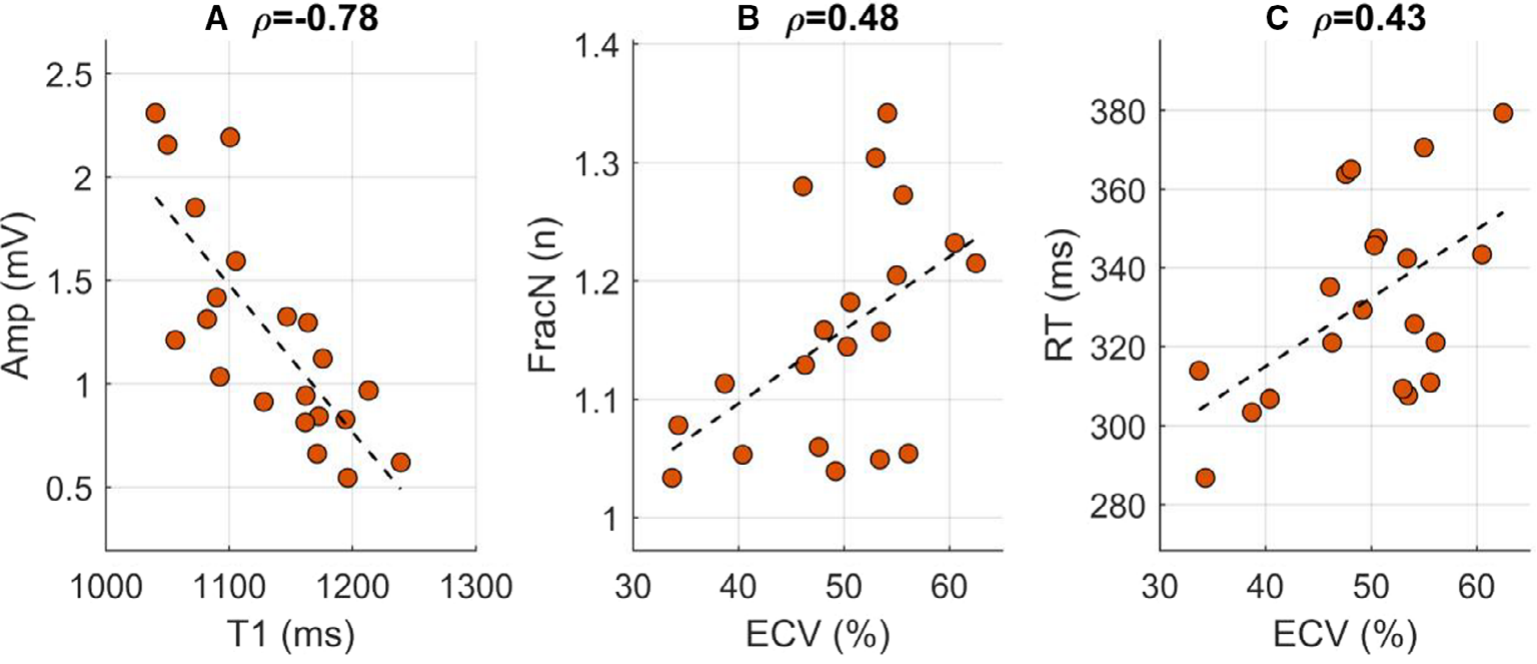
Correlations between ECGI (vertical axis) and CMR (horizontal axis) parameters, where ρ indicates the correlation coefficient. **A**, Signal amplitude (Amp) vs T1; (**B**) mean number of negative deflections (FracN) in fractionated QRS complexes vs extracellular volume (ECV); (**C**) mean repolarization time (RT) corrected for heart rate vs extracellular volume. CMR indicates cardiac magnetic resonance imaging; ECGI, ECG imaging.

Correlation coefficients between ECGI and CMR parameters are reported in Table 4. T1 was moderately correlated with ECV (ρ=0.44, *P*=0.052) and inversely correlated with total myocardial cell volume (ρ=−0.54, *P*=0.014). A very high (negative) correlation coefficient characterized the association between T1 and epicardial signal amplitude (Figure 5A, ρ=−0.78, *P*<0.001). Signal amplitude also showed a moderate correlation with total myocardial cell volume (ρ=0.48, *P*=0.021) and T2 (ρ=−0.45, *P*=0.039). ECV positively correlated with signal fractionation (Figure 5B, ρ=0.48, *P*=0.032) and showed a borderline significant correlation with repolarization time (Figure 5C, ρ=0.43, *P*=0.058).

**Table 4:** Correlation Coefficients Between CMR‐CMR and CMR‐ECGI Parameters

	ECV	T1	T2	Total cell volume	Total amyloid volume
CMR‐CMR					
ECV					
T1	0.44				
T2	−0.13	0.35			
Total cell volume	−0.15	−0.54^a^	−0.23		
Total amyloid volume	0.46^a^	−0.02	−0.17	0.16	
CMR‐ECGI					
Amp	−0.32	−0.78^a^	−0.45	0.48^a^	−0.05
Frac	0.48^a^	0.22	0.09	−0.32	0.33
mFrac	0.35	0.26	0.22	−0.44^a^	0.08
ΔAT	−0.03	−0.04	0.09	0.09	0.33
AT	0.02	−0.13	−0.09	0.41^a^	0.07
ΔRT	−0.29	−0.02	−0.03	0.18	−0.17
RT	0.43^a^	0.34	−0.20	0.22	0.29
ΔARI	−0.18	0.01	−0.20	0.12	−0.10
ARI	0.36	0.39	−0.07	0.06	0.27
G_AT_	−0.11	−0.08	0.02	0.08	0.24
G_RT_	−0.19	0.10	0.06	−0.11	−0.25

Repolarization parameters were corrected for heart rate. Parameters were calculated using anatomical segments in common with both technologies. Amp indicates mean epicardial signal amplitude; AT, RT, and ARI, mean activation, repolarization, and activation‐repolarization interval, respectively; CMR, cardiac magnetic resonance imaging; ECGI, ECG imaging; ECV, mean extracellular volume; Frac, mean number of negative deflections in fractionated QRS complexes; G_AT_ and G_RT_, spatial gradients of activation and repolarization; mFrac, maximum number of deflections in fractionated QRS complexes; ΔAT, ΔRT, ΔARI, dispersion of activation, repolarization and ARI, respectively.

a Statistically significant correlations.

### Accuracy of Automatic Classification Models

The univariate models showing higher accuracy in classifying AL and ATTR patients used T2 (accuracy=81.0%) followed by T1, Total Cell Volume, and LV mass (accuracy=71.4%) from CMR, and epicardial signal amplitude (accuracy=71.4%) from ECGI. There was no significant difference between the accuracy provided by the best CMR univariate model and that from the best ECGI univariate model (81.0% versus 71.4%, *P*=0.50). Penalized logistic regression using the least absolute shrinkage and selection operator identified several multivariate models with similar deviance. Among these, those that used the fewest features included T2+ΔRT (accuracy=85.7%) and T2+LV mass+ΔRT (accuracy=90.5%). The difference in accuracy with respect to the best univariate model was nonsignificant (*P*>0.37).

## Discussion

This study combined CMR and ECGI for the first time to simultaneously investigate the interaction between structural and EP substrates in cardiac AL and ATTR patients. In particular, the utilization of ECGI allowed noninvasive mapping of ventricular extracellular potentials for the first time, as the only 2 previous EP studies were confined to atrial mapping.^33^, ^34^ The main findings were these. (1) Cardiac amyloidosis patients have EP abnormalities that include slow and spatially heterogeneous ventricular conduction, prolonged and spatially dispersed repolarization, as well as low‐amplitude epicardial potentials. (2) Despite having lower total myocardial amyloid volume, AL patients display more notable EP abnormalities compared with ATTR patients, including lower epicardial signal voltage, higher degree of signal fractionation, and slightly higher dispersion of repolarization. (3) Signal amplitude showed a strong inverse correlation with T1, whereas both signal fractionation and repolarization time increased with ECV. (4) Univariate models based on single features from both CMR and ECGI classified AL and ATTR patients with an accuracy of 70% to 80%, and the best bivariate model included both CMR and ECGI features (T2 and ΔRT) and showed 85.7% accuracy.

These findings may have implications in identifying those patients potentially at risk of conduction disturbances and ventricular arrhythmias during active treatment. Indeed, implantable loop recorder studies show a significant incidence of complete heart block before the demise of AL patients^2^ as well as cases of ventricular tachycardia.^1^, ^3^, ^35^, ^36^

Results from surface ECG analysis showed differences between cardiac amyloid and control patients in line with previous findings.^30^, ^37^, ^38^, ^39^, ^40^, ^41^, ^42^ Interestingly, standard surface ECG parameters were not different between AL and ATTR patients, whereas ECGI was able to identify significant ventricular conduction and repolarization differences between the 2 cardiac amyloidosis types, suggesting that ECGI parameters may enable a better discrimination between AL and ATTR and may improve risk stratification for ventricular arrhythmias. Furthermore, ECGI analysis detected a significant prolongation of the ARI (a surrogate for action potential duration) and its spatial distribution in cardiac amyloidosis. This was missed by standard surface ECG analysis, which suggested that QTc prolongation could be explained by QRS widening. Combined ECGI‐CMR analysis showed that epicardial signal amplitude inversely correlated with T1 and T2, a measure of myocardial edema,^12^ and positively correlated with total myocardial cell volume. The strong inverse correlation between native T1 and epicardial signal amplitude is interesting, as native T1 is a combined signal from the intra‐ and extracellular space, and, as such, it is influenced by both amyloid deposits and myocardial edema (thought to be secondary to the light chain toxicity/high rate of amyloid deposition), the 2 main pathophysiological mechanisms linked to disease progression in AL amyloidosis.^8^, ^9^ Native T1 and epicardial signal voltage may be epiphenomena of a similar underlying process, and future studies should investigate whether their simultaneous assessment may increase prognostic accuracy.

Our finding that cardiac amyloidosis is associated with enhanced spatial gradients of activation suggests the existence of disturbances in electrical conduction at the tissue level, which was also reflected by a higher incidence and complexity of epicardial signal fractionation in cardiac amyloidosis versus control patients. A positive correlation between ECV and signal fractionation suggests a link between disturbances in electrical conduction and extracellular deposition. However, the lack of direct correlation between ECV and activation time may indicate that the interaction between ECV and conduction is complex and possibly modulated at the tissue level by the spatial distribution of amyloid deposition. Interestingly, ECV also correlated with average repolarization time, suggesting that amyloid deposition may be directly implicated in the abnormal prolongation of ventricular repolarization demonstrated in this study and previously reported in cardiac amyloidosis.^43^, ^44^ The fact that both activation‐recovery interval and its spatial dispersion were significantly longer in cardiac amyloidosis than in controls suggests that abnormal repolarization patterns may be largely independent of conduction abnormalities.

Our results show that although ATTR patients had higher amyloid volume than AL patients, the latter were characterized by more severe EP abnormalities. This supports the hypothesis that additional mechanisms beyond amyloid infiltration may contribute to the greater mortality in AL amyloidosis, such as light chain toxicity,^9^ previously proven by in vitro studies, or faster rate of amyloid deposition.^8^ In particular, ECGI data suggest that AL patients were characterized by more spatially heterogeneous conduction and repolarization than ATTR. Spatial conduction‐repolarization heterogeneity is a marker of both increased arrhythmic and pump failure risk^45^, ^46^, ^47^ and may be related to worse prognosis and a higher incidence of sudden cardiac death in AL versus ATTR patients.

Future studies based on larger cohorts are needed to determine whether this integrated analysis of both structural and electrophysiological substrates could better capture the disease complexity and provide additional insights for the development of personalized prognostic and therapeutic strategies.

### Limitations

This was an exploratory study and the assessment of the clinical impact of combining CMR and ECGI for the management of cardiac amyloidosis patients should be determined in specifically designed confirmatory studies. At this stage, classification analysis was not performed with the aim to propose and validate a specific diagnostic model but to demonstrate that ECGI and CMR provide complementary information that may be useful to better characterize AL and ATTR substrate and risk profile. Because this is the first study to have combined ECGI and CMR in cardiac amyloidosis, it was not possible to test the classification model on another data set, but standard leave‐1‐out cross validation was performed to reduce overfitting. Furthermore, the study cohort was small, and the within‐group variability of some variables was relatively high. However, the results of CMR and surface ECG analysis are in line with previous findings.^5^, ^7^, ^14^, ^30^, ^37^, ^38^, ^39^, ^40^, ^41^, ^42^ Although a similar ECV between AL and ATTR groups could indicate that patients were studied at a similar stage of the disease, it may be possible that part of the differences observed between AL and ATTR patients were caused by the disease progression at the time of the study. Recent studies have reported conflicting results about the accuracy of a commercial ECGI system,^48^, ^49^ and the utilization of noninvasively computed as opposed to invasively measured potentials in this study may have affected the results. However, in this study we have used a noncommercial system that has undergone extensive validation,^50^, ^51^, ^52^ and electrograms were carefully analyzed and revised semiautomatically as in previous studies.^25^, ^26^ Because ECGI measures epicardial potentials, correlation between ECGI and CMR parameters were investigated excluding septal segments. Although EP differences between cardiac amyloidosis and controls could be partially explained by age, all differences in ECGI parameters remained significant between age‐matched subgroups, suggesting that EP abnormalities cannot be entirely explained by age. Future studies should investigate the differences in electrophysiological parameters after adjustment for other confounders, such as medication, comorbidity, and standard cardiovascular risk factors.

## Conclusions

This study shows that cardiac amyloidosis is associated with both ventricular conduction and repolarization abnormalities, which are more pronounced in cardiac AL than in ATTR patients. This represents a first step toward the integration of structural and EP parameters for a better pathophysiological characterization and more accurate arrhythmia and sudden cardiac death risk stratification in this population.

## Sources of Funding

This study was supported by British Heart Foundation (BHF) grant FS/18/21/33447 and Royal Society Grant IE141601. ECGI maps were processed in the Rudy laboratory under support from NIH grant R01‐HL33343. Lambiase is supported by University College London Hospital (UCLH) Biomedicine NIHR and received educational grants from Medtronic. O'Regan, de Marvao, and Cook are funded by the Medical Research Council, United Kingdom. The study was supported by the Academy of Medical Sciences (SGL015/1006) and the National Institute for Health Research Biomedical Research Centre based at Imperial College Healthcare NHS Trust and Imperial College London.

## Disclosures

Dr Rudy was a cochair of the scientific advisory board and received royalties from CardioInsight Technologies. CardioInsight Technologies does not support any research conducted in Dr Rudy's laboratory. The remaining authors have no disclosures to report.

## Supporting information


**Table S1.** Patients’ Age, Sex, Etiology, and Medication
**Table S2.** 12 Lead ECG Parameters
**Table S3.** ECGI Parameters
