# Supplementary material for: Noninvasive Mapping of the Electrophysiological Substrate in Cardiac Amyloidosis and Its Relationship to Structural Abnormalities
Source: J Am Heart Assoc. 2019 Sep 9;8(18):e012097. doi: 10.1161/JAHA.119.012097 (PMC6818012; doi:10.1161/JAHA.119.012097)
Supplement: Supplementary file 1 — Table S1. Patients’ Age, Sex, Etiology, and Medication Table S2. 12 Lead ECG Parameters Table S3. ECGI Parameters [file JAH3-8-e012097-s001.pdf]

SUPPLEMENTAL MATERIAL

Table S1. Patients' age, sex, aetiology and medication.

	Sex	Age	Aetiology	Medication
1	M	85	TTR	Apixaban 5mg bd, Tamsulosin 400mcg od, Lansoprazole 15mg od, Atorvastatin 10mg od, Doxycycline 100mg bd, Furosemide 80mg bd, Spironolactone 50mg od
2	M	68	AL	Ranolazine 500mg bd, Amiodarone 100mg od, Furosemide 40mg bd, Ramipril 1.25mg od, Omeprazole 20mg od, Rivaroxaban 20mg od, Questerinhaler
3	M	77	TTR	Bisoprolol 1.25mg od, Solifenacin 10mg od, Tamsulosin 400mcg, Doxazosin 4mg od, Atorvastatin 10mg od, Amlodipine 5mg od, Fisateride 5mg od, Aspirin 75mg od, Ramipril 10mg od, Bisacodyl 10mg od
4	M	86	TTR	Rivaroxaban
5	F	65	AL	Bumetanide 3mg bd, Simvastatin 40mg od, Mirtazapine 15mg od, Zopiclone 7.5mg od
6	M	47	TTR	Furosemide, Atorvastatin, Amlodipine, Omeprazole, Glicazide, Metformin, Empagliflozin
7	M	59	AL	Bumetanide 1mg, Spironolactone 50mg od, Doxycycline 100mg bd, Rivaroxaban
8	M	73	TTR	Tildiem 200mg od, Rivaroxaban 20mg od, Ramipril 1.25mg od, Spironolactone 25mg od, Gabapentin 1 tds
9	M	80	TTR	Goserelin 10.8mg every three months, Accrete D-3
10	M	72	AL	Aspirin 75mg, Furosemide 40mg od, Valsartan 320mg od, Atorvastatin 40mg od
11	M	74	AL	Rivaroxaban, Atorvastatin, Ramipril 1.25mg, Bisoprolol 7.5mg, Lansoprazole, B12 injections, Furosemide 40mg bd
12	M	77	TTR	Furosemide 40mg od, Lansoprazole, Folica acid, Simvastatin
13	M	85	TTR	Ramipril 2.5mg, Simvastatin 40mg, Bisoprolol 2.5mg, Eplerenone 50mg, Warfarin, Lansoprazole 30mg, Furosemide 40mg, Finasteride 5mg, Tamsulosin 400mcg
14	M	82	TTR	Warfarin, Allopurinol, Candesartan, Atorvastatin
15	M	83	TTR	Furosemide 40mg bd
16	F	60	AL	Thyroxine 75mcg od, Losartan 6.25mg bd, Co-Amiloride, Furosemide 40mg od, Citalopram 10mg od, Rosuvastatin 5 mg od
17	M	85	AL	Furosemide 40mg bd, Aspirin, Atorvastatin, GTN spray, Monomil XL 60mg od, Tamsulosin 400mcg od, Acyclovir 400mg bd, Omeprazole 20mg od, Co-trimoxazole
18	M	65	AL	Ranitidine 150mg
19	M	62	AL	Valsartan 100mg bd, Levothyroxine 100mcg od, Rivaroxaban 20mg od, Rosuvastatin 5mg od, Omeprazole 20mg od, Tamsulosin 400mcg od, Glucophage 500mg bd, Furosemide 40mg od

20	M	64	AL	Apixaban, Bisoprolol, Furosemide 80mg bd, Atorvastatin
21	M	64	AL	Furosemide 120mg od, Ramipril, Simvastatin, Warfarin

Table S2. 12 lead ECG parameters.

Surface ECG Parameters	Amyloidosis (Age Matched) n=10	Controls (Age Matched) n=10	P-value	Amyloidosis (Rest of patients) n=11	P-value
Age (years)	63.00 (17.00)	64.28 (8.18)	0.520	82.00 (16.00)	**0.014**
AF (n)	1 (10.0%)	0 (0.0%)	1.0000	2 (18.2%)	1.0000
RR (ms)	852 (182.0)	929 (312.0)	0.6232	900 (187.5)	0.2047
QRS (ms)	107 (16.0)	88 (10.0)	**0.0015**	104 (35.0)	0.9437
PR (ms)	172 (32.0)	166 (28.0)	0.3446	194 (73.5)	0.3608
QTc (ms)	431 (53.6)	411 (14.7)	0.2123	452 (42.0)	0.1131
Poor R progression (n)	9 (90.0%)	0 (0.0%)	**0.0001**	8 (72.7%)	0.5865
BBB (n)	1 (10.0%)	0 (0.0%)	1.0000	2 (18.2%)	1.0000
TW Inversion (n)	5 (50.0%)	0 (0.0%)	**0.0325**	3 (27.3%)	0.3870
Amp-Limb (mV)	0.38 (0.22)	0.92 (0.33)	**0.0009**	0.40 (0.23)	0.6983
Amp-Precordial (mV)	1.04 (0.40)	1.34 (0.22)	**0.0450**	1.40 (0.51)	0.2907
Amp-Sokolow (mV)	1.05 (1.00)	2.40 (0.30)	**0.0136**	1.40 (1.02)	0.6981
Low Voltage-Limb (n)	6 (60.0%)	0 (0.0%)	**0.0108**	5 (45.5%)	0.6699
Low Voltage-Precordial (n)	1 (10.0%)	0 (0.0%)	1.0000	1 (9.1%)	1.0000
Low Voltage-Sokolow (n)	7 (70.0%)	0 (0.0%)	**0.0031**	6 (54.5%)	0.6594

Differences between the cardiac amyloidosis patients (first column) and controls (second column) matched by age. The third column shows differences between cardiac amyloidosis patients age-matched to controls and the rest of cardiac amyloidosis patients. AF: Atrial fibrillation. QTc: QT interval corrected for heart rate (Fridericia’s formula); BBB: Bundle branch block; TW: T-wave; Amp: Amplitude. Statistically significant P-values are reported in bold.

Table S3. ECGI Parameters.

ECGI Parameters	Amyloidosis (Age Matched) n=10	Controls (Age Matched) n=10	P-value	Amyloidosis (Rest of patients) n=11	P-value
Age (years)	63.00 (17.00)	64.28 (8.18)	0.520	82.00 (16.00)	**0.014**
HR (bpm)	77.46 (16.62)	67.64 (28.49)	0.970	65.05 (19.89)	0.418
Amp (mV)	1.11 (0.25)	1.96 (0.87)	**0.014**	1.60 (0.83)	0.170
Frac (n)	1.21 (0.09)	1.10 (0.13)	**0.004**	1.18 (0.18)	0.418
mFrac (n)	3.00 (2.00)	3.00 (1.00)	0.401	3.00 (1.00)	0.630
ΔAT (ms)	60.30 (16.11)	42.97 (8.30)	**0.005**	68.36 (37.48)	0.275
AT (ms)	30.46 (5.10)	25.17 (4.73)	**0.045**	30.93 (10.51)	0.647
ΔRT (ms)	168.93 (37.28)	129.29 (33.50)	**0.006**	163.23 (45.56)	0.916
RT (ms)	309.09 (31.96)	273.46 (14.26)	**0.001**	328.05 (35.58)	0.130
ΔARI (ms)	177.61 (53.43)	136.55 (36.77)	**0.026**	191.97 (63.19)	0.549
ARI (ms)	278.11 (34.47)	245.14 (12.49)	**0.007**	290.73 (22.80)	0.130
GAT (ms/mm)	0.34 (0.05)	0.24 (0.07)	**0.005**	0.39 (0.18)	0.379
GRT (ms/mm)	0.94 (0.62)	0.75 (0.21)	0.089	0.80 (0.32)	0.218

Differences between the cardiac amyloidosis patients (first column) and controls (second column) matched by age. The third column shows differences between cardiac amyloidosis patients age-matched to controls and the rest of cardiac amyloidosis patients. Amp: Mean epicardial signal amplitude; Frac: Mean number of negative deflections in fractionated QRS complexes; Frac-m: Maximum number of deflections in fractionated QRS complexes; AT, RT, ARI: Mean activation, repolarization and ARI, respectively. ΔAT, ΔRT, ΔARI: Dispersion of activation, repolarization and ARI, respectively. G_{AT} and G_{RT}: Spatial dispersion of activation and repolarization. Repolarization parameters, were corrected for heart rate. Statistically significant P-values are reported in bold.
